# Advancing Clinical Outcomes in Maternal–Fetal Medicine

**DOI:** 10.3390/jcm14124104

**Published:** 2025-06-10

**Authors:** Katarzyna Kosińska-Kaczyńska, Michał Ciebiera

**Affiliations:** 1 Department of Obstetrics, Perinatology and Neonatology, Centre of Postgraduate Medical Education, 01-813 Warsaw, Poland; 2 Second Department of Obstetrics and Gynecology, Centre of Postgraduate Medical Education, 00-189 Warsaw, Poland; mciebiera@cmkp.edu.pl; 3 Warsaw Institute of Women’s Health, 00-189 Warsaw, Poland

## 1. Introduction

Maternal–fetal medicine is a rapidly evolving field, continuously integrating innovative research findings to enhance clinical outcomes both for mothers and newborns. This Special Issue of *Clinical Outcomes in Maternal–Fetal Medicine* presents critical insights into key areas of perinatal care, including the risks associated with adolescent pregnancies, novel delivery techniques aimed at reducing neonatal morbidity, awareness of congenital infections, and neonatal outcomes in pregnancy complications and their prediction. The findings of these studies significantly contribute to evidence-based clinical decision-making and underscore the need for multidisciplinary collaboration in advancing maternal and neonatal healthcare.

## 2. Editorial

### 2.1. En Caul Cesarean Delivery: A Novel Strategy for Preterm Neonatal Protection

Pabin et al. investigated the benefits of “en caul” cesarean delivery, in which the neonate is born within the intact amniotic sac [1]. This approach may reduce mechanical trauma, umbilical cord prolapse, and the risk of neonatal intracranial hemorrhage, but its use is limited due to technical challenges in preserving membrane integrity [2]. Recent studies suggest that “en caul” delivery may offer particular advantages for extremely preterm infants with a very low birth weight by maintaining a protective intrauterine-like environment. Jin et al. reported better hemodynamic stability and lower lactate levels, indicating reduced hypoxia [3]. Despite its promise, this technique demands surgical expertise and carries maternal risks. Its broader adoption will require randomized trials to establish clear guidelines and safety profiles.

### 2.2. Awareness of Congenital Cytomegalovirus Infection: A Critical Gap in Prenatal Education

Congenital cytomegalovirus (cCMV) infection represents the most prevalent congenital viral infection globally, with incidence rates ranging from 0.58% in developed countries to as high as 5% in developing regions [4]. Bartnik et al. conducted a semi-systematic review evaluating pregnant patients’ awareness of cCMV, revealing alarmingly low knowledge levels, with awareness rates being as low as 11.4% in Ireland and up to 60% in France [5]. This knowledge gap is particularly concerning given that prenatal antiviral therapy was demonstrated to significantly reduce the vertical transmission risk [6,7]. The review highlights the effectiveness of educational interventions in improving awareness and preventive behaviors in pregnant individuals. Given the increasing availability of secondary prevention strategies, it is imperative to integrate structured cCMV education programs into routine prenatal care to enhance maternal knowledge and reduce the neonatal morbidity associated with congenital CMV infection.

### 2.3. Fetal Weight Estimation in Congenital Diaphragmatic Hernia: Revisiting Predictive Accuracy

In their study, Kuchnowska et al. investigated the reliability of standard fetal weight estimation formulae in cases of congenital diaphragmatic hernia (CDH), a condition characterized by the herniation of abdominal organs into the thoracic cavity, often leading to pulmonary hypoplasia and postnatal respiratory distress [8]. Since CDH often results in an underestimation of the abdominal circumference, concerns were raised regarding the accuracy of conventional fetal weight formulae, particularly the Hadlock formula [9]. The study compared the Hadlock and Faschingbauer formulae in a cohort of 42 CDH cases and 80 healthy controls, concluding that the Hadlock formula, when adjusted for the ultrasound-to-delivery interval, remained the most precise tool for estimating fetal weight. The findings reinforce the clinical utility of standard biometry in CDH pregnancies while emphasizing the need for individualized growth assessments and perinatal planning to optimize neonatal outcomes. This study may have implications for the future management of this disease.

### 2.4. Hemolytic Disease of the Fetus and Newborn: A Persistent Challenge in Perinatal Medicine

Drozdowska-Szymczak et al. presented a retrospective cohort study analyzing the management and treatment outcomes of hemolytic disease of the fetus and newborn (HDFN), a condition resulting from maternal antibodies attacking fetal red blood cells. Despite routine antenatal anti-D prophylaxis, a subset of affected fetuses still required intrauterine transfusions (IUTs) [10]. Among 274 neonates with HDFN, 46 required an IUT due to fetal anemia, while 228 did not. The study revealed that neonates treated with IUT exhibited higher rates of significant anemia, hyperbilirubinemia, and iron overload, as evidenced by elevated ferritin levels. In a study published recently, 97% of the neonates after IUT required admission to a neonatal intensive care unit, 88% underwent phototherapy, and 25% required rehospitalization due to anemia necessitating red blood cell transfusion [11]. This study reinforces the necessity of continued advancements in antenatal screening, immunoprophylaxis, and fetal therapy to minimize the impact of HDFN on perinatal outcomes.

### 2.5. A Multicenter, Retrospective Comparison Study of Pregnancy Outcomes According to Placental Location in Placenta Previa

Placenta previa is one of the most serious pregnancy complications, and may lead to significant maternal–fetal complications, including massive bleeding, the need for the early completion of pregnancy, and an increased risk of postpartum complications. A multicenter study (781 participants) presented by researchers from the Republic of Korea aimed to analyze the impact of placental location on the course and outcomes of pregnancy, indicating that placental location (anterior, posterior, or central) might differentiate the risk of specific complications [12]. The results suggested that patients with anterior placenta previa were more likely to experience profuse intrapartum bleeding. However, the authors’ observations mostly indicated that more caution was required in the treatment of patients with anterior placenta previa. Further research is necessary to develop precise management protocols depending on the placental location. The personalization of care based on modern imaging techniques and risk prediction may significantly reduce the incidence of complications, improving safety for both the mother and child.

### 2.6. Occurrence and Risk Factors of Cholestasis in Newborns with Hemolytic Disease—A Case–Control Study

Neonatal cholestasis is a serious condition that may occur in newborns with hemolytic disease (HDFN), leading to impaired liver function and long-term health consequences. The case–control study conducted by Drozdowska-Szymczak et al. allowed for the identification of key risk factors conducive to the development of cholestasis in this population [13]. The authors assessed the impact of the severity of hemolysis, prematurity, and exposure to exchange transfusions on the incidence of this complication. The results indicated that newborns with severe hyperbilirubinemia and low birth weight were at the highest risk of developing liver dysfunction. The risk factors for cholestasis in children with HDFN included lower gestational age at delivery, Rh and Kidd serological types of HDFN, and the need for intrauterine transfusions. Similar results were published by other authors [14]. Early diagnosis and the implementation of suitable treatment, including the use of so-called hepatoprotectors and monitoring liver parameters, may significantly improve treatment results.

### 2.7. Adolescent Pregnancy: A Comparative Insight into the Prevalence and Risks of Obstetric Complications in a Polish Cohort

Pregnancy in adolescents remains a global public health challenge, with an increased risk of complications both for young mothers and their offspring. In their narrative review, Maheshwari et al. emphasize that adolescent pregnancy is associated with significant risks for both the mother and the newborn. The most common complications include anemia, gestational hypertension, preterm birth, low birth weight, and an increased risk of admission to the neonatal intensive care unit [15]. Staniczek et al. conducted a study in a Polish cohort and showed that pregnant adolescents were more likely to develop gestational hypertension, anemia, and fetal growth disorders compared to older pregnant women [16]. In addition, premature births and a low birth weight of newborns were more common in this group, which increased the risk of neonatal complications. These results emphasize the need to implement effective educational and prevention programs tailored for young women, aimed at improving their health awareness and increasing access to prenatal care.

### 2.8. Navigating Uncertain Waters: The Role of First-Trimester Screening in Identifying Neonatal Complications

Modern prenatal medicine strongly emphasizes the early detection of potential pregnancy-related complications, which allows for the better preparation and management of mother and child care. First-trimester screening plays a key role in identifying the risk of such complications. Świercz et al. presented the data of 1164 patients who underwent first-trimester screening, including patient history, ultrasound examinations, and biochemical tests for pregnancy-associated plasma protein-A (PAPP-A) and the free beta-HCG subunit (fbHCG) [17]. They found that low concentrations of PAPP-A and the free bHCG subunit in the first trimester might be associated with poorer clinical and biochemical conditions in neonates post delivery. However, the relationship was weak and had limited predictive capability. This interesting article indicates that the early detection of abnormalities enables the implementation of suitable medical interventions, which may significantly improve health outcomes in the mother and the newborn. Regular first-trimester screening becomes the standard of prenatal care, emphasizing the importance of screening in modern obstetric practice.

### 2.9. Maternal–Fetal Complications in Renal Colic During Pregnancy: A Scoping Review

Renal colic during pregnancy presents a significant clinical challenge due to potential complications both for the mother and the fetus. Women affected by renal colic have a higher rate of perinatal complications—such as urinary tract infections and preterm birth—compared to the general population of pregnant women [18]. A scoping review by Machura et al. indicated increased risks of premature delivery, gestational hypertension, and urinary tract infections in women with renal colic [19]. Moreover, untreated renal colic may lead to intrauterine growth restriction and a low birth weight of the newborn. Early diagnosis and suitable treatment, comprising the minimization of risk to the fetus, are crucial to ensure optimal pregnancy outcomes [20,21].

## 3. Conclusions: Bridging Research and Clinical Practice

The research presented in this Special Issue underscores the multifaceted challenges in maternal–fetal medicine and the importance of applying evidence-based strategies to improve perinatal care. Addressing the unique risks associated with adolescent pregnancy, refining delivery techniques for preterm neonates, enhancing awareness of congenital infections, optimizing fetal weight estimation methodologies, and improving the management of HDFN are all pivotal in advancing maternal and neonatal health outcomes. The studies featured herein not only contribute to the growing body of literature, but also provide actionable insights that may inform clinical practice, policy-making, and future research endeavors.

The findings presented in this issue highlight the necessity of continued interdisciplinary collaboration, particularly in integrating novel research into obstetric and neonatal protocols. As maternal–fetal medicine continues to evolve, fostering innovation through robust clinical trials and translational research will be essential in shaping the future of perinatal healthcare and ensuring the best possible outcomes both for mothers and their newborns.
